# Arctigenin Suppresses Breast Cancer Growth In Vitro and In Vivo Through Subtype-Specific Multi-Targeting Activity

**DOI:** 10.3390/ijms27115055

**Published:** 2026-06-03

**Authors:** Joshua Yang, Qiongyu Hao, Ke Wu, Yahya Elshimali, Ali Andalibi, Piwen Wang

**Affiliations:** 1 Division of Cancer Research and Training, Charles R. Drew University of Medicine and Science, Los Angeles, CA 90059, USA; joshuayang@cdrewu.edu (J.Y.); qiongyuhao@cdrewu.edu (Q.H.); kewu@cdrewu.edu (K.W.); yahyaelshimali@cdrewu.edu (Y.E.); 2 Institute for Advanced Biomedical Research, Charles R. Drew University of Medicine and Science, Los Angeles, CA 90059, USA; aliandalibi@cdrewu.edu

**Keywords:** arctigenin, phytochemical, MCF-7, SKBR3, MDA-MB-231, estrogen receptor (ER), human epidermal growth factor receptor 2 (HER2), triple-negative breast cancer (TNBC), xenograft

## Abstract

Arctigenin (Arc), a novel anti-inflammatory lignan derived primarily from *Arctium lappa*, has demonstrated promising anticancer activity in multiple cancer types. This study was designed to evaluate the anticancer efficacy of Arc across distinct molecular subtypes of breast cancer in vitro and in vivo and to gain mechanistic insights into its mode of action. In vitro evaluation was conducted in estrogen-receptor-positive MCF-7, human epidermal growth factor receptor 2 (HER2)-positive SKBR3, and triple-negative MDA-MB-231 breast cancer cell lines. In vivo efficacy and safety were evaluated using female severe combined immunodeficient (SCID) mice (5–7 weeks old) bearing MCF-7 or MDA-MB-231 xenografts. Mice received daily oral gavage of Arc at 50 mg/kg body weight for 8 weeks. In vitro, Arc inhibited cell proliferation across all three breast cancer subtypes in a dose-dependent manner. PCR-array analysis of gene expression revealed that Arc targets multiple signaling molecules involved in cell proliferation, cell cycle regulation, apoptosis, migration/invasion, and drug transport, demonstrating a subtype-specific target profile. Arc induced cell-cycle arrest at the G2/M phase in MCF-7 cells and at G0/G1 in MDA-MB-231 cells, accompanied by significant induction of apoptosis in both cell lines. Migration assays further demonstrated marked inhibition of wound closure in Arc-treated cells. In vivo, Arc treatment significantly inhibited tumor growth in both xenograft models, decreased Ki67 expression, and produced no overt toxicity. In summary, Arc exhibits potent anticancer activity against distinct breast cancer subtypes through multi-targeting mechanisms. Given the heterogeneity of breast cancer, Arc appears to be a promising candidate for further preclinical investigation.

## 1. Introduction

Breast cancer remains the most commonly diagnosed cancer and the second leading cause of cancer-related death among women in the United States, accounting for an estimated 15.5% of new cancer cases and 6.9% of all cancer deaths in 2025 [1]. Despite significant advancements in early detection, treatment strategies, and overall survival rates, breast cancer continues to present major clinical challenges due to its heterogeneity, therapy resistance, and metastatic potential [2]. The molecular heterogeneity of breast cancer is characterized by varying expression levels of estrogen receptor (ER), progesterone receptor (PR), and human epidermal growth factor receptor 2 (HER2). Breast cancer is commonly classified into luminal A (ER+, PR+, HER2−), luminal B (ER+, PR+, HER2+/−), HER2-enriched (ER−, PR−, HER2+), and triple-negative breast cancer (TNBC; ER−, PR−, HER2−) subtypes [2].

TNBC accounts for approximately 15–20% of breast cancer cases [3]. Due to the absence of ER, PR, and HER2 expression, TNBC does not respond to standard hormone therapies or HER2-targeted treatments, making this subtype particularly aggressive and associated with the poorest prognosis among breast cancer subtypes [3]. The primary systemic treatment for TNBC remains conventional chemotherapy, typically including anthracyclines, cyclophosphamide, and taxanes [3]. Although recent advances in immunotherapy, particularly immune checkpoint inhibitors for PD-L1-positive tumors, have expanded therapeutic options, TNBC continues to carry a higher risk of recurrence and visceral metastasis compared with other breast cancer subtypes [3]. Additionally, immunotherapies for TNBC are often associated with immune-related adverse effects, including endocrinopathies and gastrointestinal toxicities [3]. Given the limitations of conventional cancer therapies, such as chemotherapy and radiotherapy, including treatment-related toxicity and the development of drug resistance, there is an increasing need for alternative, natural-based compounds that offer multi-targeting therapeutic benefits with minimal adverse effects to address these complex challenges.

**Figure 1:**
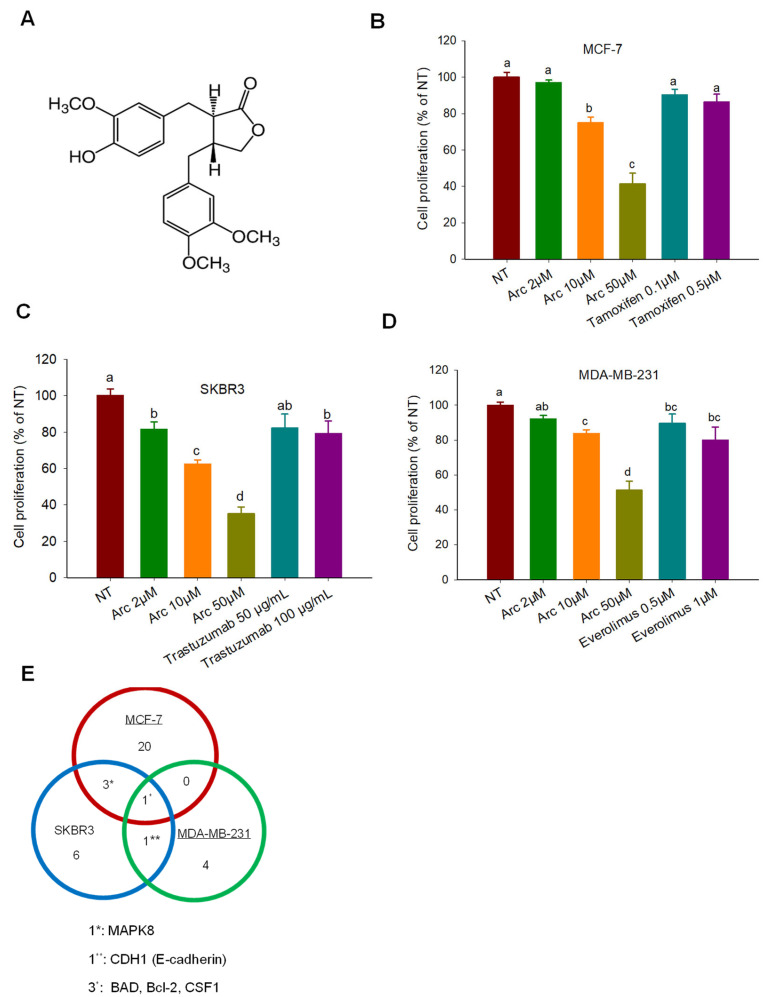
Arctigenin inhibits the proliferation of breast cancer cells and modulates gene expression. (**A**) Chemical structure of arctigenin (Arc). Luminal A MCF-7 (**B**), HER2-enriched SKBR3 (**C**), and triple-negative MDA-MB-231 (**D**) cells were treated with Arc at desired concentrations, positive control clinical drugs, or DMSO vehicle control (non-treated, NT) for 48 h. Cell proliferation was assessed using the ATP-based CellTiter-Glo^®^ Luminescent Cell Viability Assay. Proliferation is expressed as a percentage relative to NT controls. (**E**) A PCR-array assay of the mRNA expression of genes involved in breast cancer proliferation, apoptosis, migration/invasion, among other cancer-related processes. The number of genes significantly modulated by Arc treatment was indicated with each of the subtypes, including the number of overlapping genes. Lists of these genes are provided in the Table 1. Data are presented as mean ± SD. Different letters denote a significant difference between groups, *p* < 0.05.

Arctigenin (Arc, chemical structure in Figure 1A) is a bioactive lignan derived mainly from the seeds of *Arctium lappa*, a medicinal herb traditionally used in Chinese medicine to treat inflammation-related diseases [4]. Preclinical studies by our group and others have demonstrated that Arc exhibits potent anticancer activity by suppressing angiogenesis, inhibiting tumor growth, and inducing apoptosis in multiple cancer types, including prostate [5,6,7], colorectal [8], and pancreatic [9] cancers. The multi-targeting activity of Arc may contribute to more durable cancer control, as cancers often harbor numerous genetic alterations, dysregulated pathways, and extensive signaling crosstalk that promote chemoresistance [10]. We further demonstrated in animal models that the potent anticancer activity of Arc is associated with its much higher bioavailability compared with other phytochemicals, such as green tea polyphenols [5,11]. In addition, preclinical evidence suggests that Arc may exert selective cytotoxic effects against malignant breast cancer cells while sparing normal mammary epithelial cells, indicating a favorable therapeutic index [12]. However, the therapeutic efficacy of Arc across distinct molecular subtypes of breast cancer and its underlying mechanisms of action remain insufficiently characterized.

In the present study, we investigated the therapeutic potential of Arc across different breast cancer subtypes in vitro and in animal models and explored the molecular targets underlying its effect on cell growth inhibition, cell cycle regulation, apoptosis induction, and migration suppression. Tumor proliferation was evaluated by Ki67 expression, a widely used marker of cell proliferation and tumor aggressiveness that is associated with breast cancer progression and higher tumor grade across multiple breast cancer subtypes [13]. To our knowledge, this is among the first in vivo evaluations of Arc in ER-positive MCF-7 xenograft models and among the first comparative in vivo assessments across distinct breast cancer subtypes. By targeting cancer cells with diverse cellular contexts, Arc shows promise as a therapeutic candidate capable of addressing the heterogeneity of breast cancer.

## 2. Results

### 2.1. Arc Exhibits Significant Antiproliferative Effects in All Three Subtypes of Breast Cancer Cells

MCF-7, SKBR3, and MDA-MB-231 cells were treated with Arc at a series of concentrations or a vehicle control for 48 h to assess antiproliferative activity. Arc significantly inhibited cell proliferation in a dose-dependent manner across all three cell lines (Figure 1B–D). IC_50_ values were determined using a range of Arc concentrations (1, 2, 5, 10, 20, 50, and 100 µM). The calculated IC_50_ values were 11.6 µM for MCF-7 cells, 10.8 µM for SKBR3 cells, and 14.1 µM for MDA-MB-231 cells. When compared with clinical drugs at clinically relevant concentrations, the results suggest that Arc has the potential to achieve efficacy comparable to or greater than that of the selected clinical drugs across the tested breast cancer subtypes (Figure 1B–D), given its favorable safety profile, as we demonstrated before [11].

PCR-array analysis revealed that Arc was able to modulate the expression of multiple genes involved in distinct breast cancer subtypes, demonstrating a subtype-specific target profile (Figure 1E; Table 1). Genes were considered significantly regulated if they exhibited a ≥2-fold change in mRNA expression and a *p* value < 0.05 relative to the control. The target gene set was largest in MCF-7 cells, intermediate in SKBR3 cells, and smallest in MDA-MB-231 cells. The modulated genes are related to various cellular processes, including cell cycle regulation (e.g., *CDKN1A*, *CDKN1C*), apoptosis (e.g., *BAD*, *BCL2*), migration/invasion (e.g., *CDH1*, *EGFR*), and drug transport/efflux (e.g., *ABCB1*). Several genes were commonly targeted by Arc across the cell lines, including *BAD*, *BCL2*, and *CSF1* in MCF-7 and SKBR3 cells; *CDH1* in SKBR3 and MDA-MB-231 cells; and *MAPK8* in all three cell lines (Figure 1E). A full list of Arc-modulated genes in each cell line is provided in Table 1.

### 2.2. Arc Induces Cell Cycle arrest in MCF-7 and MDA-MB-231 Cells

To validate whether Arc-induced antiproliferative effects were associated with cell cycle arrest and apoptosis, MCF-7 and MDA-MB-231 cells were treated with 10 µM or 50 µM Arc or vehicle control for 48 h and analyzed for both cell cycle distribution and Annexin V-based apoptosis.

**Figure 2:**
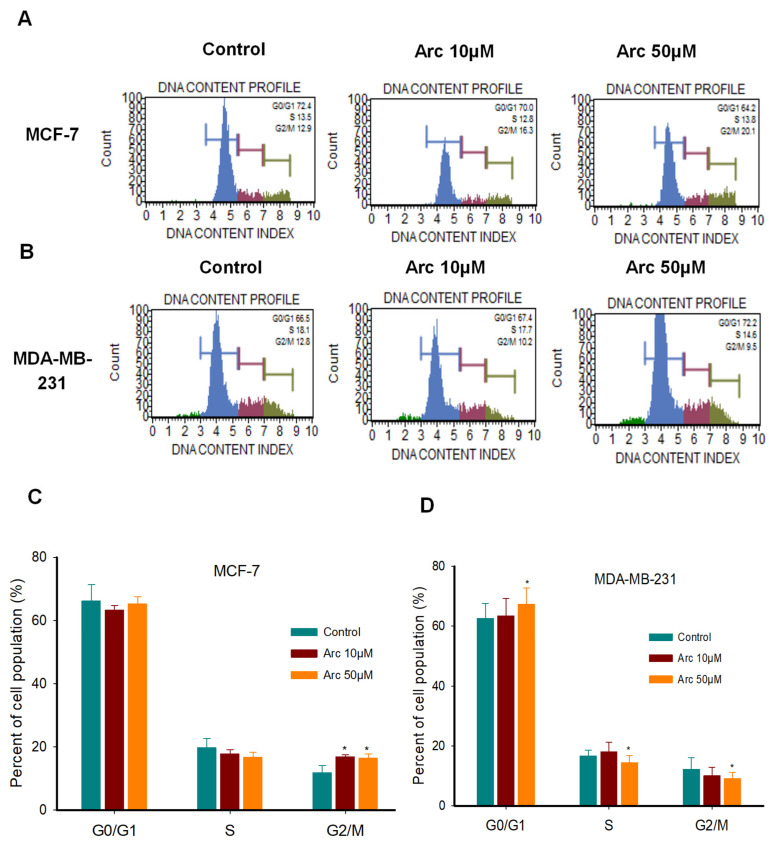
Arctigenin induces G2/M-phase arrest in MCF-7 and G0/G1 arrest in MDA-MB-231 breast cancer cells. MCF-7 (**A**,**C**) and MDA-MB-231 (**B**,**D**) were treated with 10 µM or 50 µM Arc for 48 h and analyzed by Muse^®^ cell-cycle flow cytometry. Gated histograms (**A**,**B**) and quantified phase distributions (**C**,**D**) show G2/M accumulation in MCF-7 and G0/G1 accumulation in MDA-MB-231 cells, accompanied by reduced S-phase populations. Blue peaks in A and B represent G0/G1 phase, red peaks S phase, and green peaks G2/M phase. Data are presented as mean ± SD. * *p* < 0.05 vs. control.

In MCF-7 cells, treatment with Arc at both concentrations significantly induced G2/M-phase cell cycle arrest (Figure 2A,C). In contrast, Arc induced a shift toward G0/G1 arrest in MDA-MB-231 cells (Figure 2B,D). A dose-dependent decrease in the G2/M-phase population and an increase in the G0/G1 population were observed, with 50 µM Arc producing a statistically significant shift compared to the DMSO control (Figure 2D).

### 2.3. Arc Induces Apoptosis in MCF-7 and MDA-MB-231 Cells

**Figure 3:**
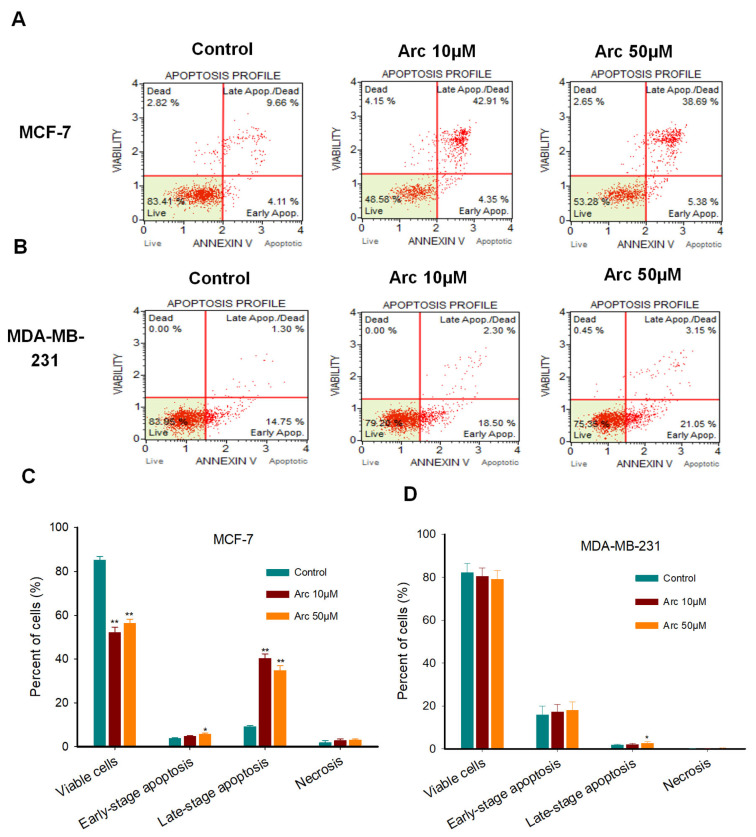
Arctigenin induces apoptosis in MCF-7 and MDA-MB-231 breast cancer cells. Representative Muse^®^ Annexin V & Dead Cell Kit Scatterplot of viable, early apoptotic, late apoptotic, and necrotic populations following 48 h treatment with 10 µM or 50 µM Arc in MCF-7 (**A**) or MDA-MB-231 (**B**) cells. Quantification shows Arc significantly increases late-stage apoptosis and reduces cell viability in MCF-7 cells (**C**). Arc induces a dose-dependent increase in late-stage apoptosis in MDA-MB-231 cells (**D**). Data are presented as mean ± SD. * *p* < 0.05, ** *p* < 0.01 vs. control.

Representative scatterplot gating and results from the apoptosis assay are shown following 48 h treatment with 10 µM or 50 µM Arc in MCF-7 (Figure 3A) or MDA-MB-231 (Figure 3B) cells. In MCF-7 cells, Arc significantly reduced the viable cells from approximately 85% in controls to ~52% at 10 µM and ~62% at 50 µM Arc (Figure 3C). This decrease in viability corresponded with a significant increase in late-stage apoptosis, rising from ~8% in controls to ~40% at 10 µM and ~35% at 50 µM Arc (Figure 3C). Early apoptosis also increased slightly but remained below 10%, with a significant increase in the percentage of cells under the 50 µM Arc treatment (Figure 3C).

In MDA-MB-231 cells, apoptosis analysis confirmed a dose-dependent decrease in viable cells and an increase in both early and late apoptotic populations (Figure 3D). Specifically, 50 µM Arc significantly increased the proportion of cells undergoing late-stage apoptosis compared to the DMSO control (Figure 3D).

### 2.4. Arc Reduces Wound Closure in Wound-Healing Assay

**Figure 4:**
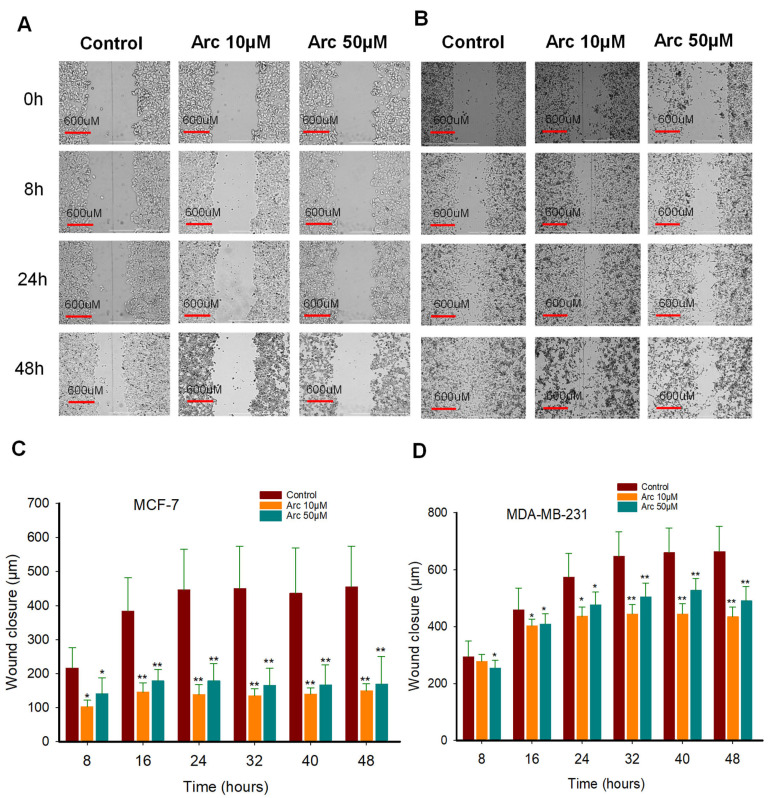
Arctigenin inhibits migration of MCF-7 and MDA-MB-231 cells in wound-healing assays. Representative images of scratch wounds over 48 h in Arc-treated vs. control in MCF-7 (**A**) and MDA-MB-231 (**B**) monolayers. Quantified wound closure over time demonstrates significantly reduced migration in MCF-7 (**C**) and MDA-MB-231 (**D**) cell lines treated with 10 µM or 50 µM Arc. Data are presented as mean ± SD. * *p* < 0.05, ** *p* < 0.01 vs. control.

A wound-healing (scratch) assay was performed to evaluate the effects of Arc on cell migration in MCF-7 (Figure 4A) and MDA-MB-231 (Figure 4B) breast cancer cells. Both cell lines exhibited significantly reduced wound closure and impaired migratory capacity following Arc treatment (Figure 4A,B). Initial wound width distances at time zero were not significantly different among treatment groups, confirming consistent scratch size. In MCF-7 cells, treatment with 10 µM and 50 µM Arc resulted in significantly reduced wound closure after 8 h compared with the DMSO control (Figure 4C). Both Arc-treated groups continued to exhibit significantly reduced wound closure relative to the control group throughout the remainder of the experiment (Figure 4C), suggesting impaired migratory capacity. A similar pattern was observed in MDA-MB-231 cells. Treatment with 50 µM Arc significantly reduced wound closure after 8 h compared with the DMSO control (Figure 4D). After 16 h, both Arc-treated groups exhibited significantly reduced wound closure compared with the control group (Figure 4D).

### 2.5. Arc Inhibits Tumor Growth in SCID Mouse Xenograft Models

**Figure 5:**
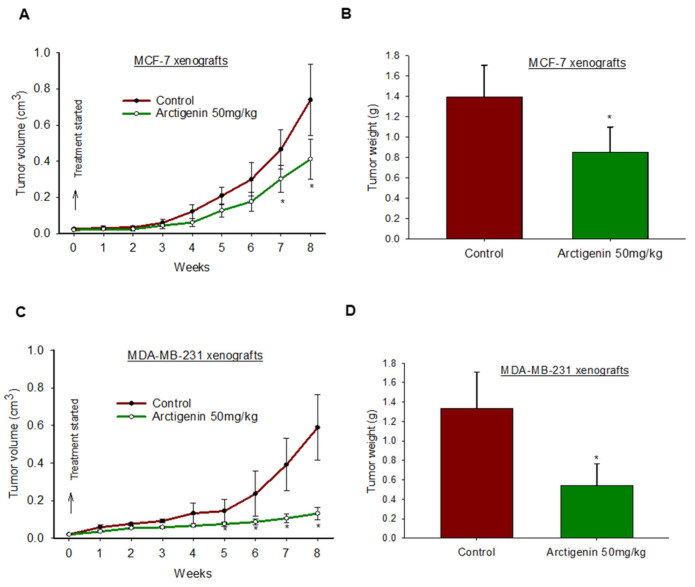
Arctigenin suppresses tumor growth in SCID mice bearing MCF-7 and MDA-MB-231 xenografts. Severe Combined Immunodeficiency (SCID) mice were subcutaneously implanted with either MCF-7 (**A**,**B**) or MDA-MB-231 (**C**,**D**) human breast cancer cells and treated daily by oral gavage with Arc at 50 mg/kg body weight or vehicle control for 8 weeks (*n* = 5 per group). (**A**,**C**) Tumor volume was monitored weekly using digital calipers in MCF-7 and MDA-MB-231 xenografts, respectively. (**B**,**D**) Tumor weight was measured at the experimental endpoint following euthanasia. Arc treatment significantly reduced tumor growth and final tumor weight in both models. Data are presented as mean ± SD. * *p* < 0.05 vs. control (two-tailed Welch’s *t*-test).

To validate the in vitro anticancer effects of Arc, a SCID mouse xenograft model was used to assess its tumor-inhibitory efficacy in vivo. Arc treatment significantly suppressed tumor growth in mice bearing MCF-7 or MDA-MB-231 xenografts (Figure 5). At a daily dose of 50 mg/kg body weight, Arc significantly reduced tumor volume in MCF-7 xenografts beginning at week 7 and in MDA-MB-231 xenografts as early as week 5 (Figure 5A,C).

Following euthanasia at the 8-week endpoint, tumors were excised and weighed. Arc-treated mice exhibited a significant reduction in final tumor weight by approximately 40% in MCF-7 tumors and 60% in MDA-MB-231 tumors compared to controls (Figure 5B,D). These findings confirm the antitumor activity of Arc in both hormone-receptor-positive and triple-negative breast cancer models.

### 2.6. Arctigenin Reduces Tumor Cell Proliferation In Vivo

**Figure 6:**
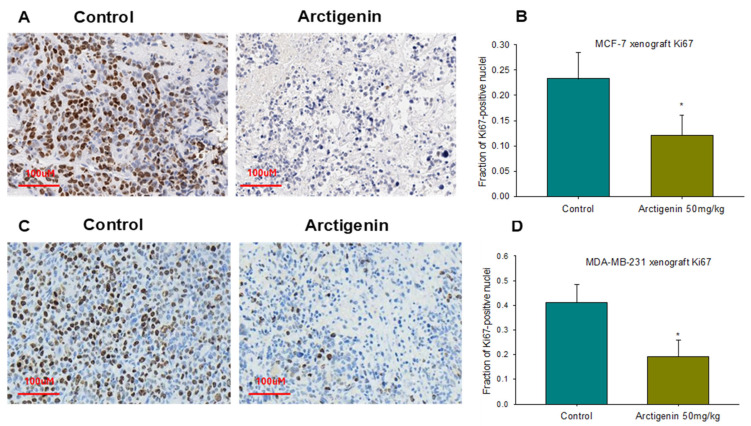
Arctigenin reduces tumor cell proliferation in xenograft tumors as shown by Ki67 immunohistochemistry. Representative immunohistochemical staining of Ki67 in tumor tissues from control and Arc-treated MCF-7 xenograft mice (**A**), and quantitative analysis of Ki67-positive nuclei relative to the total number of nuclei shows a significant decrease in the Arc-treated group compared to control (**B**). Representative images from control and Arc-treated MDA-MB-231 xenograft mice (**C**) and quantitative analysis of Ki67-positive nuclei relative to total nuclei (**D**). Data are presented as mean ± SD. * *p* < 0.05 vs. control (two-tailed Welch’s *t*-test).

To assess the antiproliferative activity of Arc in vivo, tumor tissues were harvested from SCID mice bearing MCF-7 or MDA-MB-231 xenografts and stained for the proliferation marker Ki67 (Figure 6). Compared to control tumors, Arc-treated tumors exhibited a marked reduction in Ki67 expression (Figure 6A,C), with quantitative analysis showing a decrease of about 50% in MCF-7 xenografts (Figure 6B) and 60% in MDA-MB-231 xenografts (Figure 6D), indicating a significant inhibition of tumor cell proliferation.

### 2.7. Arctigenin Shows No Adverse Effects on Body Weight, Food/Water Intake, or Liver Histology In Vivo

**Figure 7:**
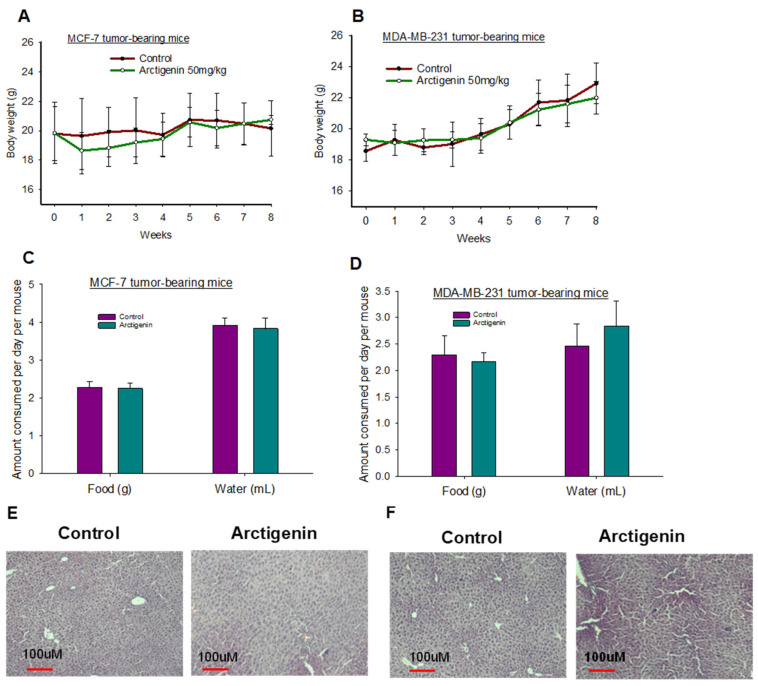
Arctigenin does not alter body weight, food & water consumption, or liver morphology in tumor-bearing mice. Body weight (**A**,**B**) and food/water consumption (**C**,**D**) in MCF-7 (**A**,**C**) and MDA-MB-231 (**B**,**D**) tumor-bearing mice treated with Arc or the vehicle were monitored weekly. No significant differences were observed between control and Arc groups. Liver tissues from MCF-7 (**E**) and MDA-MB-231 (**F**) mice were processed for H&E staining, showing no evidence of toxicity, inflammation, necrosis, or metastasis.

Arc treatment did not cause significant changes in body weight (Figure 7A,B) or food/water consumption (Figure 7C,D) in either MCF-7 or MDA-MB-231 xenograft mice. To evaluate potential liver toxicity or metastatic spread, liver tissues were examined using H&E staining (Figure 7E,F). Pathological evaluation of liver tissues revealed normal hepatic architecture with no signs of toxicity, inflammation, necrosis, or metastasis (Figure 7E,F). Overall, no overt signs of toxicity or liver histopathological alterations were observed under the conditions tested.

## 3. Discussion

This study demonstrates that Arc dose-dependently inhibits cell proliferation, induces cell cycle arrest and apoptosis, and reduces migratory potential in different molecular subtypes of human breast cancer cells. Arc may exert anti-proliferative effects that are comparable to or even stronger than those of the selected clinical drugs at clinically relevant concentrations, as suggested by the current results. However, it should be noted that these clinical drugs primarily exert cytostatic rather than acute cytotoxic effects [14,15,16], and their antitumor activity may become more pronounced with prolonged treatment. Our in vivo studies using both MCF-7 and MDA-MB-231 xenograft models further confirmed the significant tumor-inhibitory efficacy and favorable safety profile of Arc.

The anticancer activity of Arc is associated with its multi-targeting properties, as revealed by our PCR-array analysis of breast cancer-related genes. Since a cancer often harbors numerous genetic mutations/dysfunctions and extensive signaling crosstalk, therapeutic approaches directed at a single or limited number of targets often fail to produce sustained efficacy, eventually leading to the development of chemoresistance [17]. This limitation is common to most current therapeutic drugs [17]. Natural compounds like Arc target multiple events and signaling pathways involved in tumorigenesis, potentially enabling systemic control of this disease [18]. Furthermore, our PCR-array analysis demonstrated that Arc has a subtype-specific target profile (Figure 1E, Table 1), supporting its capacity to selectively modulate dysregulated cellular signaling pathways. Selective targeting has also been reported for other phytochemicals, including green tea polyphenols, which may underlie their favorable safety profiles in normal cells and their broad-spectrum cytotoxicity against multiple cancer types [19]. Considering the substantial heterogeneity of breast cancer, even within the same tumor [20], the multi-targeting activity of Arc is both crucial and promising for effective disease control.

A dysregulated cell cycle is a hallmark of oncogenesis that can lead to uncontrolled and unchecked proliferation, leading to cancer formation and progression [21]. Modulating cell cycle checkpoints represents an important therapeutic mechanism [21]. Our cell cycle analysis revealed that Arc induced cell cycle arrest in both MCF-7 and MDA-MB-231 cells, though the patterns differed between the two cell lines. Arc triggered G2/M-phase arrest in MCF-7 cells while inducing G0/G1-phase arrest in MDA-MB-231 cells. This observation is consistent with our PCR-array results, which showed that *CDKN1A* (*p21*) mRNA expression increased in MCF-7 cells following Arc treatment. Elevated *p21* levels may subsequently inhibit cyclin-dependent kinases (CDKs), particularly the CDK1–cyclin B complex, thereby preventing entry into mitosis (M phase) and resulting in G2/M cell cycle arrest [22]. In contrast, Arc increased *BRCA2* expression, rather than *p21*, in MDA-MB-231 cells. *BRCA2* is a tumor suppressor gene essential for DNA repair, and its mutation or genetic silencing is particularly prevalent in TNBC, leading to the accumulation of DNA damage and increased genomic instability [23]. The Arc-induced increase in *BRCA2* expression in MDA-MB-231 cells may have triggered cell cycle arrest, specifically in the G0/G1 phase, allowing time for DNA repair or, if repair fails, the initiation of apoptosis [24]. Furthermore, increased *CDH1* (*E-cadherin*) expression in MDA-MB-231 cells in response to Arc treatment may contribute to G1-phase arrest by promoting the degradation of mitotic cyclins, particularly cyclin B, and associated CDKs [25].

Arc also robustly activated apoptotic pathways in all three breast cancer cell lines, consistent with its antiproliferative effects (Figure 1). The pro-apoptotic effect of Arc was further validated in MCF-7 and MDA-MB-231 cells using the flow cytometry assay (Figure 3). Apoptosis is a tightly regulated form of programmed cell death and serves as a critical mechanism for the elimination of cancer cells [26]. Arc-induced apoptosis was associated with the modulation of multiple signaling molecules and was dependent on the cellular context [27]. Upregulation of *MAPK8* was observed across all three breast cancer subtypes. *MAPK8* is a central component of stress-activated signaling pathways and frequently exerts pro-apoptotic effects [28]. Additionally, Arc treatment increased the expression of the pro-apoptotic gene *BAD* and decreased the expression of *BCL-2*, the key anti-apoptotic regulator [29], in both MCF-7 and SKBR3 cells. It should be noted that MCF-7 cells are caspase-3-deficient; therefore, apoptosis in this model may involve alternative caspase-dependent or caspase-independent mechanisms [30]. In MDA-MB-231 cells, in addition to increased *BRCA2* and *CDH1* expression, which may promote apoptosis via cell cycle arrest, another noteworthy gene with reduced expression by Arc treatment is *ABCB1*, also known as *MDR1* [31]. This gene encodes P-glycoprotein, an efflux pump implicated in multidrug resistance in cancer cells [31]. By modulating the intracellular concentration of pro-apoptotic compounds, *ABCB1* can indirectly influence the cell’s susceptibility to apoptosis [31].

**Figure 8:**
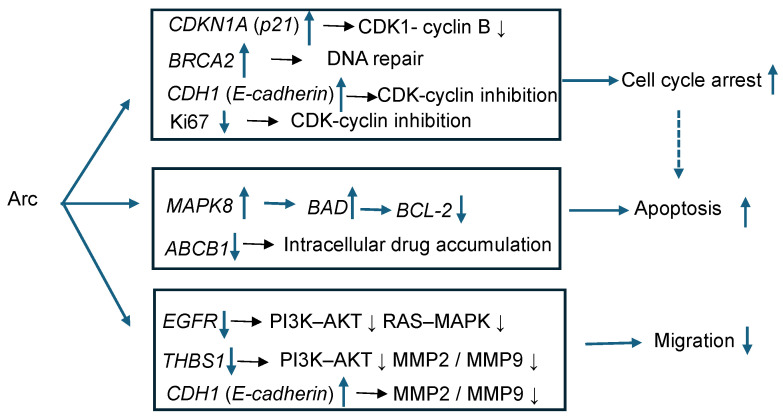
Schematic summary of key molecules that may play a central role in mediating the anticancer effects of Arc in breast cancer. Arrows indicate Arc-induced up- or down-regulation, and the dashed line represents a possible pathway. Blue arrows indicate findings from the present study, whereas black arrows represent putative downstream molecules based on the literature.

Migration assays further supported Arc’s anti-metastatic potential. Arc significantly reduced the migratory potential of both MCF-7 and MDA-MB-231 cells, as evidenced by wound-healing assays (Figure 4), suggesting impaired motility and cell–cell coordination. Our PCR-array analysis suggests that multiple molecules may contribute to the inhibitory effects of Arc on cell migration. In MCF-7 cells, Arc treatment was associated with reduced expression of epidermal growth factor receptor (*EGFR*) and thrombospondin 1 (*THBS1*). EGFR promotes cell migration by activating key signaling pathways, such as MAPK and PI3K/Akt, which regulate cytoskeletal dynamics, cell adhesion, and gene expression [32]. THBS1, an extracellular matrix protein, facilitates tumor cell migration and invasion and is highly expressed in many aggressive cancers, including breast cancer, where it is associated with advanced disease stage, lymph node involvement, brain metastasis, and therapeutic resistance [33]. It should be noted that MCF-7 cells are inherently less migratory than MDA-MB-231 cells due to their more epithelial phenotype. Therefore, migration-related findings in MCF-7 cells should be interpreted with caution [34]. In contrast, in MDA-MB-231 cells, Arc treatment significantly increased the expression of *CDH1* (*E-cadherin*). E-cadherin is a critical mediator of cell–cell adhesion, and its expression restricts tumor cell detachment and dissemination. Loss of CDH1 is frequently observed in breast cancers and is generally associated with poor prognosis [35]. Prior studies have linked Arc to the downregulation of matrix metalloproteinases (MMP-2/-9) and heparanase, enzymes crucial for extracellular matrix remodeling and cancer progression [36]. Collectively, modulation of these pathways by Arc may underlie the impaired cell migration observed in our assays. Figure 8 summarizes the potential mechanisms underlying the anti-breast cancer effects of Arc.

To further validate the anticancer potential of Arc, an in vivo study was conducted using a SCID mouse xenograft model. This well-established model is widely used to investigate tumor growth, pathogenesis, and therapeutic efficacy with human cancer cells [37]. Due to technical difficulties in establishing stable SKBR3 xenograft tumors in SCID mice at the time of the study, we focused on MCF-7 and MDA-MB-231 models for additional mechanistic investigations and in vivo validation. In the present study, Arc markedly suppressed tumor growth in SCID mice implanted with either MCF-7 or MDA-MB-231 cells. Immunohistochemical analysis of tumor tissues from both xenograft models further confirmed the antiproliferative activity of Arc, as evidenced by a 50–60% reduction in Ki-67 expression in MCF-7 and MDA-MB-231 tumors (Figure 6B,D). A reduction in Ki67 is a strongly positive prognostic factor, indicating effective treatment [38]. These in vivo findings are consistent with our in vitro results, corroborate previous reports [12], and underscore the tumor-suppressive potential of Arc.

Importantly, Arc exhibited a favorable safety profile following oral administration. No significant changes in body weight or food and water consumption were observed in Arc-treated mice compared to the control (Figure 7A–D). Histopathological examination of liver tissues revealed no evidence of toxicity, inflammation, necrosis, or metastasis, indicating that Arc is well tolerated at the tested dose (Figure 7E,F). These findings are particularly relevant for future translational studies evaluating Arc as a potential therapeutic lead.

Several limitations of the present study should be considered. First, although transcriptional changes associated with treatment were identified, protein-level validation of key targets and signaling pathways is needed to confirm the functional relevance of these molecular alterations. Second, results of the wound-healing assay should be interpreted cautiously, as wound closure may be influenced not only by cell migration but also by treatment-induced changes in cell proliferation and apoptosis, particularly at later time points. In the present study, early time points (8 and 16 h) more accurately reflect migratory effects, while later time points may be partially influenced by proliferation-related changes. Third, the relatively small sample size used in the animal experiments may limit the extent of interpretation of the in vivo findings. Therefore, the in vivo work should be considered exploratory and confirmatory within the scope of a preliminary preclinical study. Finally, the toxicity assessment was limited and primarily based on body weight monitoring and liver histopathology; more comprehensive evaluations, including hematological, biochemical, and multi-organ toxicity analyses, are warranted in future studies. In addition, pharmacokinetic analyses were not conducted in the current breast cancer models, and thus the systemic exposure, tissue distribution, and tumor accumulation of Arc remain to be fully characterized.

## 4. Materials and Methods

### 4.1. Cell Line and Culture Conditions

Human breast cancer cell lines MCF-7 (luminal A), SKBR3 (HER2-enriched), and MDA-MB-231 (triple-negative subtype) were purchased from ATCC (Manassas, VA, USA). Cells were maintained in Dulbecco’s Modified Eagle Medium (DMEM) containing 10% fetal bovine serum, penicillin (100 IU/mL), and streptomycin (100 µg/mL). Cultures were incubated at 37 °C in a humidified atmosphere with 5% CO_2_. All cells used in this study were passaged fewer than 8 times. Routine screening for mycoplasma contamination was performed using the MycoStrip^®^ Mycoplasma Detection Kit (InVivoGen, San Diego, CA, USA).

### 4.2. Cell Proliferation Assay

MCF-7, SKBR3, and MDA-MB-231 cells were seeded in opaque-walled 96-well plates at a density of 6 × 10^3^ cells/well. The cells were treated with 2 µM, 10 µM, or 50 µM Arc (Sigma-Aldrich, St. Louis, MO, USA) or a volume-matched DMSO vehicle control (0.5% in medium) for 48 h. Clinically used drugs for different breast cancer subtypes were included as positive controls: tamoxifen (estrogen receptor antagonist) for MCF-7 cells, trastuzumab (Herceptin; HER2 receptor inhibitor) for SKBR3 cells, and everolimus (mTOR inhibitor) for MDA-MB-231 cells at circulating concentrations achievable following clinically relevant dosing [39,40,41]. Cell proliferation was assessed using the CellTiter-Glo^®^ 2.0 Luminescent Cell Viability Assay (Promega Corporation, Madison, WI, USA), which measures intracellular Adenosine Triphosphate (ATP) levels. Antiproliferative effects were expressed as percentage inhibition relative to the control.

### 4.3. PCR-Array Assay of mRNA Expression in Subtypes of Breast Cancer Cells

MCF-7, SKBR3, and MDA-MB-231 cells were treated with Arc at 10 µM or the vehicle control for 24 h. Total RNA was isolated from adherent cells using the miRNeasy Mini Kit (Qiagen, Valencia, CA, USA). Expression profiling of 84 genes associated with breast cancer initiation and progression was conducted using the RT^2^ Profiler™ Human Breast Cancer PCR Array (Qiagen) according to the manufacturer’s protocol. Briefly, extracted RNA was reverse-transcribed into first-strand cDNA with the RT^2^ First Strand Kit (Qiagen). The resulting cDNA was combined with RT^2^ SYBR Green Master Mix and subjected to quantitative real-time PCR (qRT-PCR) analysis. Gene expression data were normalized using the ΔCt method with GAPDH as a housekeeping gene. All experiments were performed in triplicate.

### 4.4. Cell Cycle Assay

Cell cycle distribution was analyzed using the Muse^®^ Cell Cycle Kit (Cytek Biosciences, Fremont, CA, USA). MCF-7 and MDA-MB-231 cells were seeded in 100 mm diameter Petri dishes at 1 × 10^6^ cells per plate. After 48 h, cells were treated with 10 µM or 50 µM Arc or the DMSO control for an additional 48 h. Attached cells were harvested via trypsinization, fixed in 70% ice-cold ethanol, and stored at −20 °C. Fixed cells (1 × 10^6^) were divided into 200 µL aliquots, centrifuged at 300× *g* for 5 min, and resuspended in 200 µL of Cell Cycle Reagent. Samples were incubated at room temperature (RT) in the dark for 30 min and analyzed using the Guava^®^ Muse^®^ Cell Analyzer (Cytek Biosciences). The experiment was repeated three times.

### 4.5. Apoptosis Analysis

Apoptosis was measured using the Muse^®^ Annexin V & Dead Cell Kit (Cytek Biosciences). MCF-7 and MDA-MB-231 cells were seeded in 100 mm diameter Petri dishes at 1 × 10^6^ cells per plate. After 48 h, the cells were treated with 10 µM or 50 µM Arc or the DMSO control for an additional 48 h. Treated attached cells (1 × 10^5^ per sample) were collected in 100 µL of culture media. A positive control was prepared by treating DMSO-control cells with 50 µM etoposide and incubating them at 37 °C for 30 min. Each sample was mixed with 100 µL of Annexin V reagent and incubated at RT in the dark for 20 min before analysis on the Guava^®^ Muse^®^ Cell Analyzer (Cytek Biosciences). The experiment was repeated three times.

### 4.6. Wound-Healing Assay

MCF-7 and MDA-MB-231 cells were seeded in 96-well plates at a density of 1 × 10^5^ cells per well. Upon reaching confluence, a scratch was introduced using the Agilent BioTek AutoScratch device (Agilent Technologies, Santa Clara, CA, USA). The cells were washed and treated with 100 µL of medium containing 10 µM or 50 µM Arc or a DMSO volume-matched control. There were 6 replicates for each group. Plates were placed in the BioTek Cytation 1 Cell Imaging Multimode Reader (Agilent Technologies), and images were captured every 2 h over a 48 h period. Image analysis was conducted using Gen5 3.13 Software (Agilent Technologies). Wound closure was calculated by subtracting the wound width at each time point from the baseline wound width.

### 4.7. In Vivo Antitumor Activity Evaluation in Xenograft Mouse Models

All animal studies were approved by the Institutional Animal Care and Use Committee (IACUC) at Charles R. Drew University of Medicine and Science and The Lundquist Institute (IACUC Project #CDU 22451). Female Severe Combined Immunodeficient (SCID) mice (5–7 weeks old; The Jackson Laboratory, Sacramento, CA, USA) were maintained under a 12 h light/dark cycle with ad libitum access to food and water. Mice were subcutaneously (SC) injected with 1 × 10^6^ MCF-7 or MDA-MB-231 cells. The MCF-7-injected mice received a subcutaneous implant of an estradiol pellet (0.72 mg, Innovative Research of America, Sarasota, FL, USA) one week before injection to mimic a sustained estrogen environment conducive to tumor growth. One week post-injection, when tumors reached a volume of approximately 10 mm^3^, mice were randomized using a simple randomization method into two groups (*n* = 5 per group, calculated based on results from our previous Arc study in prostate cancer xenograft models [11]) and treated with either Arc (50 mg/kg body weight daily; Bolise Co., Limited, Xiamen, China) or the vehicle control (2% DMSO in corn oil) via oral gavage (with a volume of approximately 100 µL) for 8 weeks. Baseline tumor volumes were comparable across groups prior to treatment initiation. Tumor volumes were measured weekly using digital calipers and calculated as length × width × height × 0.5236 [39]. Maximum tumor size did not exceed institutional ethical limits (1.5 cm in diameter). Body weight and general health status were monitored regularly to assess potential toxicity. Humane endpoints were applied according to institutional guidelines, including >20% body weight loss (body weight was measured once per week), tumor size > 1.5 cm in diameter, ruffled and/or matted fur, hypothermia, lethargy, hunched posture, inability to eat or drink, severe medical conditions that cannot be controlled with appropriate therapy, tumor interference with normal gait or movement, torticollis or barrel rolling, sudden pain or distress that cannot be controlled with analgesics, sedatives or tranquilizers, and skin ulceration over the tumor. Food and water consumption were measured weekly by recording the amounts provided and remaining, with intake calculated as the difference between the two. Values were then normalized to the number of days and the number of mice per cage (five mice per cage).

No animals were excluded from the analysis. At study termination, mice were euthanized by exsanguination following anesthesia with isoflurane, in accordance with approved protocols. No mice reached humane endpoints prior to the study endpoint. Tumor and liver tissues were harvested for immunohistochemistry and pathological evaluation.

### 4.8. Immunohistochemical Tissue Staining and Analysis

Tumor and liver tissues were fixed in 10% phosphate-buffered formalin and embedded in paraffin. Sections (4 µm thick) were cut using a Leica RM2255 Microtome (Leica Biosystems, Deer Park, IL, USA) and mounted on positively charged slides. After deparaffinization and rehydration, antigen retrieval was performed in citrate buffer (pH 6.0) at 95 °C for 20 min. Tumor tissues were stained with primary monoclonal Ki67 antibody (1:200 dilution, DAKO North America Inc., Carpineteria, CA, USA) for proliferation analysis. The slides were counterstained with hematoxylin. Slides were scanned with the ScanScope AT (Aperio Technologies Inc., Vista, CA, USA) and analyzed digitally using Definiens’ Tissue Studio (Definiens Inc., Parsippany, NJ, USA) across the entire tissue section area. A nuclear detection module with preset thresholds was used to quantify Ki67 staining. Necrotic areas were excluded from analysis to ensure that only viable tumor regions were assessed. Liver tissues were stained with hematoxylin and eosin (H&E) to assess tissue pathology. Investigators were blinded during immunohistochemical analyses and liver histopathological assessments.

### 4.9. Statistical Analysis

All data are expressed as mean ± standard deviation (SD). Statistical analysis was performed using GraphPad Prism 9 (GraphPad Software Inc., San Diego, CA, USA) and SPSS version 24 (IBM, Chicago, IL, USA). For comparisons between two groups, unpaired two-tailed Student’s *t*-tests were used. For analyses involving more than two groups, one-way ANOVA followed by post hoc multiple comparison tests was applied. For time-course data, including tumor growth curves, body weight, food and water intake, and wound-healing assays, statistical significance was assessed using two-way repeated-measures ANOVA to evaluate the effects of treatment, time, and their interaction. Where appropriate, mixed-effects models were considered for missing values or unequal variance structures. Outliers were evaluated using Grubbs’ test with caution due to the small sample size, and no outliers were identified or excluded from the current analyses. A *p*-value < 0.05 was considered statistically significant.

## 5. Conclusions

Arc exhibits potent anticancer activity against subtypes of breast cancer cells. Arc suppresses cell proliferation, disrupts cell cycle progression, induces apoptosis, and impairs migratory ability. Furthermore, Arc significantly reduced tumor growth in vivo without inducing overt toxicity under the tested conditions. Overall, these findings highlight Arc as a promising candidate for further mechanistic and preclinical validation of its therapeutic efficacy in breast cancer.

## Figures and Tables

**Table 1:** Modulation of gene expression by Arc in subtypes of breast cancer cells.

Cell Line	Up-Regulated	Fold Change	*p*-Value	Down-Regulated	Fold Change	*p*-Value
MCF-7	*BAD*	2.41	0.001	*APC*	0.42	0.013
	*CDKN1A*	3.86	0.000	*ATM*	0.32	0.003
	*CDKN1C*	2.91	0.003	*BCL2*	0.31	0.002
	*CSF1*	2.35	0.023	*CDH13*	0.44	0.021
	*GRB7*	2.28	0.015	*CST6*	0.10	0.000
	*JUN*	9.19	0.000	*EGFR*	0.47	0.032
	*MAPK8*	3.39	0.006	*GSTP1*	0.24	0.007
	*RASSF1*	2.60	0.005	*HIC1*	0.48	0.015
	*SERPINE1*	2.25	0.026	*IGF1*	0.20	0.000
				*IGFBP3*	0.37	0.003
				*KRT5*	0.45	0.025
				*PGR*	0.30	0.012
				*RARB*	0.14	0.002
				*SLIT2*	0.33	0.008
				*THBS1*	0.29	0.001
SKBR3	*BAD*	5.24	0.001	*ADAM23*	0.29	0.009
	*BRCA1*	2.52	0.011	*BCL2*	0.43	0.016
	*CDH1*	2.62	0.026	*CDH13*	0.49	0.023
	*CSF1*	2.08	0.032	*GLI1*	0.40	0.007
	*ESR2*	2.23	0.012			
	*ID1*	2.45	0.009			
	*MAPK8*	15.22	0.000			
MDA-MB-231	*BRCA2*	2.54	0.017	*ABCB1*	0.37	0.006
	*CDH1*	2.61	0.005			
	*MAPK8*	2.71	0.003			
	*MUC1*	2.85	0.013			
	*SRC*	2.62	0.021			

ABCB1, ATP-binding cassette, sub-family B (MDR/TAP), member 1. ADAM23, ADAM metallopeptidase domain 23; APC, Adenomatous polyposis coli; ATM, Ataxia telangiectasia mutated; BAD, BCL2-associated agonist of cell death; BCL2, B-cell CLL/lymphoma 2; BRCA1, Breast cancer 1, early onset; BRCA2, Breast cancer 2, early onset; CDH1, Cadherin 1, type 1, E-cadherin (epithelial); CDH13, Cadherin 13, H-cadherin (heart); CDKN1A, Cyclin-dependent kinase inhibitor 1A (p21, Cip1); CDKN1C, Cyclin-dependent kinase inhibitor 1C (p57, Kip2); CSF1, Colony stimulating factor 1 (macrophage); CST6, Cystatin E/M; EGFR, Epidermal growth factor receptor; ESR2, Estrogen receptor 2 (ER beta); GLI1, GLI family zinc finger 1; GRB7, Growth factor receptor-bound protein 7; GSTP1, Glutathione S-transferase pi 1; HIC1, Hypermethylated in cancer 1; ID1, Inhibitor of DNA binding 1, dominant negative helix-loop-helix protein; IGF1, Insulin-like growth factor 1 (somatomedin C); IGFBP3, Insulin-like growth factor binding protein 3; JUN, Jun proto-oncogene; KRT5, Keratin 5; MAPK8, Mitogen-activated protein kinase 8; MUC1, Mucin 1, cell surface associated; PGR, Progesterone receptor; RARB, Retinoic acid receptor, beta; RASSF1, Ras association (RalGDS/AF-6) domain family member 1; SERPINE1, Serpin peptidase inhibitor, clade E (nexin, plasminogen activator inhibitor type 1), member 1; SLIT2, Slit homolog 2 (Drosophila); SRC, V-src sarcoma (Schmidt-Ruppin A-2) viral oncogene homolog (avian); THBS1, Thrombospondin 1.

## Data Availability

The original contributions presented in this study are included in the article. Further inquiries can be directed to the corresponding author.

## Abbreviations

ABCB1, ATP-binding cassette, sub-family B (MDR/TAP), member 1; Arc, arctigenin; ATP, adenosine triphosphate; BAD, BCL2-associated agonist of cell death; BCL2, B-cell CLL/lymphoma 2; BRCA2, breast cancer gene 2; CDH1, cadherin 1, type 1; CDKN1A, cyclin-dependent kinase inhibitor 1A; CDKN1C, cyclin-dependent kinase inhibitor 1C; CDKs, cyclin-dependent kinases; CSF1, Colony stimulating factor 1; DMSO, dimethyl sulfoxide; EGFR, epidermal growth factor receptor; ER, estrogen receptor; H&E, hematoxylin and eosin; HER2, Human Epidermal growth factor Receptor 2; MAPK8, Mitogen-activated protein kinase 8; PR, progesterone receptor; SCID, severe combined immunodeficiency; SD, standard deviation; THBS1, thrombospondin 1; TNBC, triple-negative breast cancer.
